# p53-independent ibrutinib responses in an *Eμ-TCL1* mouse model demonstrates efficacy in high-risk CLL

**DOI:** 10.1038/bcj.2016.41

**Published:** 2016-06-10

**Authors:** H J Lee, M Gallardo, H Ma, X Zhang, C A Larsson, A Mejia, M J Hornbaker, Y Qi, X Su, L R Pageon, A Quintas-Cardama, S M Post

**Affiliations:** 1 Department of Lymphoma and Myeloma, The University of Texas MD Anderson Cancer Center, Houston, TX, USA; 2 Department of Leukemia, The University of Texas MD Anderson Cancer Center, Houston, TX, USA; 3 Department of Genetics, The University of Texas MD Anderson Cancer Center, Houston, TX, USA; 4 Department of Biostatistics, The University of Texas MD Anderson Cancer Center, Houston, TX, USA; 5 Department of Veterinary Medicine and Surgery, The University of Texas MD Anderson Cancer Center, Houston, TX, USA

## Abstract

Deletion of the short-arm of chromosome 17 (17p-) is one of the most critical genetic alterations used in chronic lymphocytic leukemia (CLL) risk stratification. The tumor suppressor *TP53* maps to this region, and its loss or mutation accelerates CLL progression, hampers response to chemotherapy and shortens survival. Although florescent *in situ* hybridization analyses for 17p deletions are routinely performed during clinical diagnoses, p53 mutational status is often unexamined. Given the limited clinical data that exists for frontline treatment of patients with CLL harboring *TP53* mutations, there is a need to understand the biology of CLL with *TP53* mutations and identify treatment strategies for this subset of patients. Herein, we used a CLL mouse model (*Eμ-TCL1*) harboring one of the most common *TP53* hot-spot mutations observed in CLL (p53^R172H^, corresponding to p53^R175H^ in humans) to evaluate its impact on disease progression, survival, response to therapy and loss of the remaining wild-type *Trp53* allele following ibrutinib treatment. We show that ibrutinib was effective in increasing survival, activating cellular programs outside the p53 pathway and did not place selective pressure on the remaining wild-type *Trp53* allele. These data provide evidence that ibrutinib acts as an effective treatment for aggressive forms of CLL with *TP53* mutations.

## Introduction

Chronic lymphocytic leukemia (CLL) is the most common type of lymphoid malignancy in the western world.^1^ Chemoimmunotherapy represents the standard therapy for the majority of CLL patients, as this treatment modality typically offers durable responses and excellent overall survival benefits.^2^ However, a subset of CLL patients with deletion of the short-arm of chromosome 17 (17p-) are refractory to chemoimmunotherapy, resulting in poor outcomes. A major reason for these poor outcomes is the loss of a key tumor suppressor gene, *TP53*, which resides at this locus and whose loss allows leukemic cells to survive when exposed to chemotherapy.^3, 4^ Recent clinical studies in CLL have shown that in addition to *TP53* deletion, mutations in the p53 pathway also result in poor outcomes.^5^ Even though p53 mutations have been implicated in exacerbating leukemic phenotypes in CLL, mutations in this gene are not commonly examined in clinical settings. As such, we have an extremely limited understanding of how these alterations impact CLL progression or responses to therapeutic interventions in an *in vivo* setting.

Given the lack of durable response to traditional treatment approaches in 17p- CLL, there is a great need to identify novel pathways that can be therapeutically exploited. The identification of B-cell receptor (BCR) activation as a key oncogenic driver has revolutionized our understanding of and approach to treating B-cell malignancies.^6^ Ibrutinib, a Bruton's tyrosine kinase inhibitor, has shown the ability to downregulate the oncogenic BCR signaling pathway and significantly improve clinical responses.^7, 8^ Although there has been no standard treatment option for CLL patients carrying a 17p-, a recent phase II clinical trial demonstrated the efficacy of ibrutinib in a high-risk/refractory CLL patient population that harbors p53 aberrations.^9^ However, due to the limited number of patients with p53 mutations (as opposed to frank deletion of the *TP53* gene), this trial cannot fully allow us to determine how p53 mutations directly impact CLL progression or responses to ibrutinib-based therapy. Thus, even though ibrutinib was recently approved by the Food and Drug Administration for the treatment of *de novo* 17p-deleted CLL, it remains unknown how, and to what extent, ibrutinib confers a survival benefit in p53-mutated CLL. Therefore, a systematic evaluation of the efficacy of ibrutinib in an *in vivo* setting of *de novo* p53-mutant CLL is warranted.

Further complicating our understanding of the impact that p53 mutations have on CLL progression is the fact that we currently do not routinely examine *de novo* CLL patients for point mutations in the p53 gene. Thus, we often do not know whether a patient harbors a p53 mutation or carries a significant clonal population with a p53 mutation at diagnosis. This deficiency results in a lack of clinical trials addressing this highly susceptible patient population and makes evaluating the impact of p53 mutations on therapeutic responses extremely challenging. Therefore, developing an *in vivo* model system that mimics the genetics of this high-risk patient population is critical. To this end, we generated a preclinical CLL mouse model (*Eμ-*TCL1)^10^ that harbors a functionally inactivating mutation in the p53 DNA-binding domain (R172H)^11^ that corresponds to one of the most common hot-spot mutations (R175H) observed in patients with CLL^12, 13^ Thus, this model represents an ideal *in vivo* platform in which to evaluate this subtype of high-risk CLL.

Herein, we examine the efficacy of ibrutinib as a frontline therapeutic modality in a mouse model of p53-mutant CLL. In this report, we show that ibrutinib increases survival regardless of p53 status through its inhibition of the BTK pathway. In addition, ibrutinib did not significantly alter the expression of p53-dependent pathways, and, most critically, did not place undue selective pressure on the remaining wild-type *Trp53* alleles to undergo loss of heterozygosity (LOH). These results suggest that ibrutinib is an effective treatment option for patients with *de novo* CLL carrying a *TP53* mutation.

## Materials and methods

### Generation of *Eμ-TCL1* and *Eμ-TCL1;p53*^*R172H/+*^ cohorts

The generation of *Eμ-TCL1* and *p53*^*R172H/+*^ mice has been previously reported.^10, 11^ To generate *Eμ-TCL1;p53*^*R172H/+*^ cohorts, *p53*^*R172H/+*^ mice were crossed with *Eμ-TCL1* mice. Mice were backcrossed five generations with C57BL/6 mice to obtain a >95% similarity. Genotypes were determined via PCR analysis, using DNA extracted from the tails of mice and the following primer sets: *Eμ-TCL1*: forward; 5′-GCCGAGTGCCCGACACTC-3′ and reverse; 5′-CATCTGGCAGCAGCTCGA-3′ and *p53*^*R172H/+*^: forward; 5′-ACCTGTAGCTCCAGCACTGG-3′ and reverse; 5′-ACAAGCCGAGTAACGATCAGG-3′. All animals were maintained according to the animal facility guidelines, and animal experiments were performed under approved protocols using MD Anderson Cancer Center and IACUC guidelines.

### Flow cytometry

Flow cytometry was performed using cells isolated from the peripheral blood of *Eμ-TCL1* and *Eμ-TCL1;p53*^*R172H/+*^ mice. Peripheral blood was collected from both untreated and treated animals at indicated time points. Briefly, erythrocytes from the peripheral blood were lysed using red blood cell lysis buffer (Becton Dickenson, San Jose, CA, USA) and ~1 × 10^6^ mononuclear cells were washed to create single-cell suspensions. Cells were re-suspended in binding buffer (Becton Dickenson) and incubated (30 min at 4 °C) with α–CD19-PECy5, α–CD5-PECy7 and α–AnnexinV-APC antibodies. Approximately 100 000 cells were acquired per sample using the Gallios flow-cytometer and software (Beckman Coulter, Indianapolis, IN, USA). All data were analyzed with FlowJo software (version 8.8.6; TreeStar, Ashland, OR, USA).

### Pathologic analyses

Mouse spleens, lymph nodes and livers obtained at necropsy were fixed in 10% formalin, paraffin embedded, sectioned and stained with hematoxylin and eosin. Morphologic analyses were performed by board-certified veterinary pathologists in the Department of Veterinary Medicine at MD Anderson Cancer Center.

### RNA-sequence analysis

Malignant B-cells were isolated from leukemic burdened spleens of *Eμ-TCL1* and *Eμ-TCL1;p53*^*R172H/+*^ mice by mechanical disruption.^14^ Isolated lymphocytes were placed in culture media (RPMI 1640 supplemented with fetal bovine serum and penicillin/streptomycin) and immediately treated with either ibrutinib (5 μM) or vehicle (DMSO). Twenty-four hours post-treatment, cells were collected and total RNA was isolated. Genotypic- and treatment-specific changes in RNA expression were determined by RNA sequencing on an Illumina platform (San Diego, CA, USA) in the MD Anderson Genotyping Core Facility. Genes were annotated using GENCODE annotation database (Version M2, GRCm38, Ensembl 73), downloaded from the GENCODE project. Aligned reads were summarized at the gene level using HT seq-count. Differential expression was performed using read count filtering, normalization, dispersion estimation and identification of differential expression were carried out using Bioconductor package EdgeR (Melbourne, VIC, Australia; www.bioconductor.org) in the MD Anderson Bioinformatics Core Facility. Low-expressing genes (<0.5 counts per million reads per sample) were filtered out and not further evaluated. Normalized expression files were analyzed with gene set enrichment analysis software (Boston, MA, USA; http://software.broadinstitute.org/gsea/index.jsp). The signal-to-noise test was applied to evaluate differential expression between dichotomous populations. Pathways with false-discovery rates <0.2 and normalized enrichment scores >1.9 were accepted as significantly differentially expressed. Positive core enrichment was used to define genes that contributed to the significance of the pathways. Raw sequence data is available from the NIH/GEO database: http://www.ncbi.nlm.nih.gov/geo/query/acc.cgi?acc=GSE76183.

### Ibrutinib treatment

*Eμ-TCL1* and *Eμ-TCL1;p53*^*R172H/+*^ cohorts were monitored daily for clinical signs of disease status. At 8 months of age, mice were weighed and treated with ibrutinib dissolved in double-distilled water (25 mg/kg per day, Selleck Chemicals, Houston, TX, USA)^15^ or vehicle (double-distilled water) via oral gavage daily until moribund. Mice were killed when moribund and diseased organs were harvested. Tissues were either placed in 10% formalin for histopathologic analysis or flash-frozen for molecular analyses. For short-term treatment, *Eμ-TCL1* and *Eμ-TCL1;p53*^*R172H/+*^ mice were treated with 25 mg/kg per day of ibrutinib via oral gavage for 5 days. Mice were killed 24 h after final treatment and tissues were harvested as above.

### Western blotting

Protein lysates were prepared from diseased splenic and lymph node tissues of treated and untreated *Eμ-TCL1* and *Eμ-TCL1;p53*^*R172H/+*^ mice in NP-40 lysis buffer supplemented with protease and phosphatase inhibitors as previously done.^16^ Fifty micrograms of soluble protein was separated by SDS-PAGE and transferred to PVDF membranes. After blocking with 5% milk in phosphate-buffered saline plus 0.1% Tween 20 (PBST) for 1 h at room temperature, membranes were incubated with either α-BTK (Cell Signaling Technology, Davers, MA, USA; 1:1000 dilution), α-ERK (Cell Signaling Technology, 1:1000 dilution), α-pBTK-(Tyr223) (Cell Signaling Technology, 1:1000 dilution), or α-pERK(Thr202/Tyr204) (Cell Signaling Technology, 1:1000 dilution) antibodies at 4 °C overnight. Membranes were then washed with PBST and incubated with α-rabbit horseradish peroxidase-conjugated secondary antibodies and visualized with ECL plus (GE Bioscience, Pittsburgh, PA, USA).

### Loss of heterozygosity

LOH of the wild-type *Trp53* allele in the *Eμ-TCL1;p53*^*R172H/+*^leukemias was determined as previously described.^17, 18^ Briefly, primer sets corresponding to exon 5 (forward) and intron 6 (reverse) were used to amplify exons five and six of the *Trp53* gene, which harbors the genetically engineered point mutation and, thus, provided a rapid and robust method to examine *Trp53* LOH. PCR amplicons were purified, sequenced and then analyzed for changes in mutational burden using Chromas software (Chromas, South Brisbane, QLD, Australia). LOH was calculated by determining the percent change in wild-type and mutant *Trp53* allelic frequency distribution.

### Statistical analysis

Analyses were performed using GraphPad Instat software (GraphPad Software, La Jolla, CA, USA). Significance was determined by Mann–Whitney's *U*-tests or Fisher's exact test for comparisons between two groups. Survival curves were plotted using the Kaplan–Meier method and compared by the log-rank (Mantel–Cox) test using GraphPad Prism 6.0 (GraphPad Software). All *P*-values were two sided and the level of statistical significance was set at <0.05.

## Results

### *Eμ-TCL1;p53*^*R172H*/+^ recapitulates the poor survival outcomes observed in human CLL patients with *TP53* mutations and does not alter the natural course of disease progression

**Figure 1:**
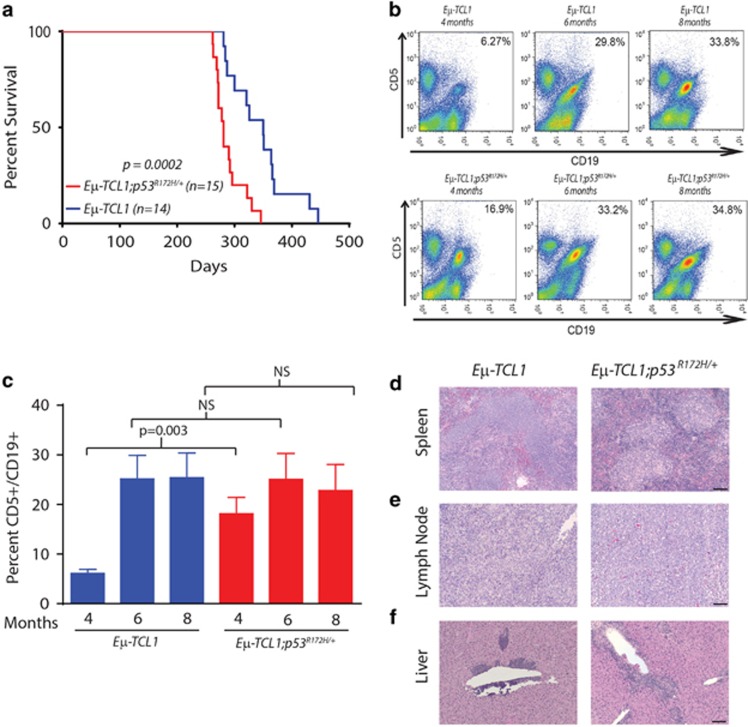
Mutant p53 results in reduced survival, but phenotypically similar disease progression in *Eμ-TCL1* mice. (**a**) Kaplan–Meier curves of *Eμ-TCL1;p53*^*R172H/+*^ and *Eμ-TCL1* mice. (**b**) Flow cytometry data demonstrating the CD5^+^/CD19^+^ immunophenotypes in the peripheral blood of *Eμ-TCL1;p53*^*R172H/+*^ and *Eμ-TCL1* mice at 4, 6 and 8 months of age. Cells were gated by their expression of CD5, CD19 and CD5/CD19. (**c**) Bar graph representing the percent of CD5^+^/CD19^+^ cells in the peripheral blood of *Eμ-TCL1;p53*^*R172H/+*^ (*n=*6) and *Eμ-TCL1* (*n=*6) mice at 4, 6 and 8 months of age. NS represents non-significant values (*P*>0.05) (**d**–**f**) Hematoxylin and eosin staining of tumor burdened spleens, lymph nodes, livers of *Eμ-TCL1*;*p53^R172H/+^* and *Eμ-TCL1* mice. The scale bar represents 100 microns.

To examine the impact that mutant p53 has on disease progression and response to targeted therapies in CLL, we generated *Eμ-TCL1* mice carrying one copy of the R172H hot-spot mutation (corresponding to the R175H mutation observed in human cancers). Using this compound mutant mouse model, we observed a significant reduction in survival of the *Eμ-TCL1;p53*^*R172H*/+^ cohort compared with mice in the *Eμ-TCL1* cohort (median 281 days versus 350 days, Figure 1a, *P*=0.0002). Although it was anticipated that mice harboring one mutant *Trp53* allele would have poorer survival, we did not observe significant differences in phenotypic disease presentation. Flow cytometry analyses revealed that the percentage of CD5^+^/CD19^+^ B-cells was similar between *Eμ-TCL1* and *Eμ-TCL1;p53*^*R172H*/+^ mice at both 6 and 8 months of age (Figures 1b and c). However, *Eμ-TCL1;p53*^*R172H*/+^ mice did present with an earlier onset of CD5^+^/CD19^+^ cells in the peripheral blood at 4 months of age, consistent with a more aggressive disease due to alteration in the p53 mutational status (Figures 1b and c). Even though there were subtle differences in disease onset at an early age, histologic analyses revealed that monomorphic mature B-cells infiltrated and ablated the normal architecture in the spleens (Figure 1d), lymph nodes (Figure 1e) and extravasated into liver parenchyma (Figure 1f) regardless of the p53 status. Together, these results demonstrate that the *Eμ-TCL1;p53*^*R172H*/+^ mouse model accelerates disease progression, but does not alter the immunophenotype or the natural presentation of the disease.

### Impaired activation of antiproliferative and apoptotic pathways in *Eμ-TCL1;p53*^*R172H*/+^ mice

**Figure 2:**
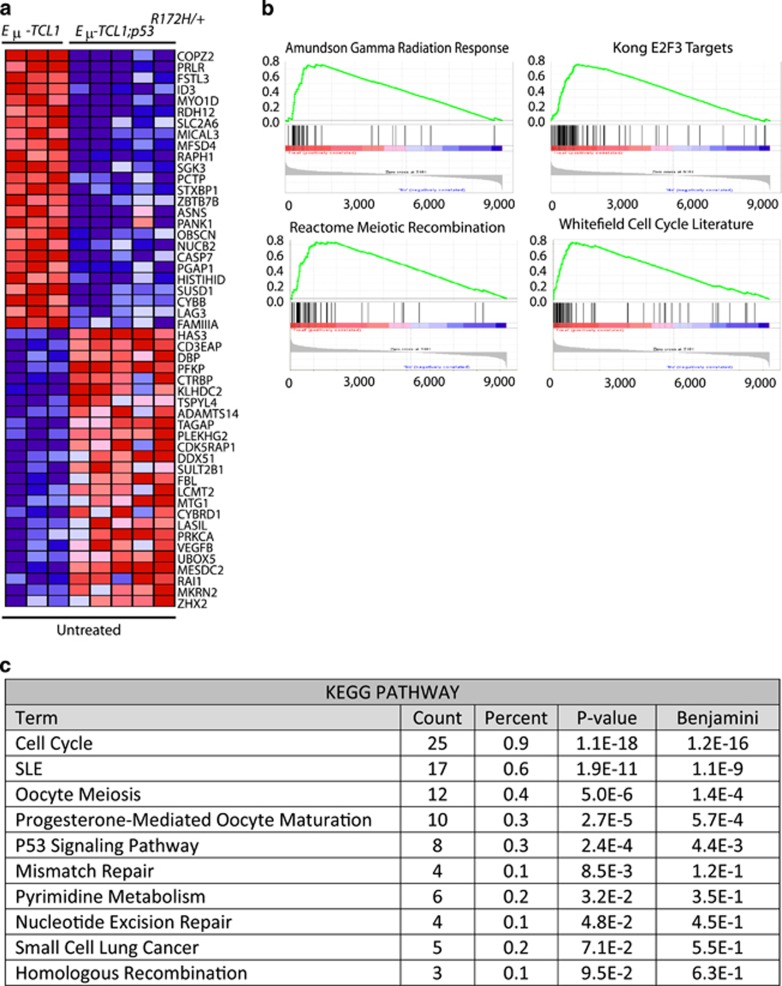
Differential gene expression profiling between B-cells from *Eμ-TCL1* and *Eμ-TCL1;p53*^*R172H/+*^ mice. (**a**) Heat map of hierarchical clustering of gene expression profiles between B-cells from *Eμ-TCL1* and *Eμ-TCL1;p53*^*R172H/+*^ mice. (**b**) Gene set enrichment analysis enrichment plots of differentially expressed gene sets from B-cells isolated from *Eμ-TCL1* and *Eμ-TCL1;p53*^*R172H/+*^ mice. Reactome Meiotic Recombination, normalized enrichment score (NES)=2.39 and false-discovery rate (FDR)=0.075; Amundsom Gamma Radiation Response, NES=2.014 and FDR=0.198; Kong E2F3, NES=1.99 and FDR=0.192; and Whitefield Cell Cycle Literature, NES=1.97, FDR=0.200. (**c**) KEGG-pathway analyses of differentially regulated biological pathways in B-cells from *Eμ-TCL1* and *Eμ-TCL1;p53*^*R172H/+*^ mice.

To understand the difference in survival between these cohorts and determine the molecular consequences of harboring a mutant *Trp53* allele in *Eμ-TCL1* mice, we isolated splenic B-cells from mice of each cohort and performed RNA-Seq analyses. Next, we compared expression changes through gene set enrichment analysis. These analyses demonstrated significant changes in gene expression profiles between the B-cells isolated from *Eμ-TCL1* and *Eμ-TCL1;p53*^*R172H*/+^ mice (Figure 2a). In addition, pathway analyses revealed that numerous p53-dependent cell cycle, DNA damage and apoptotic programs were significantly altered between these cohorts (Figures 2b and c). Together, these results indicate that the loss of one wild-type *Trp53* allele in combination with overexpression of TCL1 significantly attenuated the activation of p53-mediated pathways that directly prevent or delay disease progression.

### Ibrutinib improves survival in *Eμ-TCL1* mice regardless of p53 mutational status

**Figure 3:**
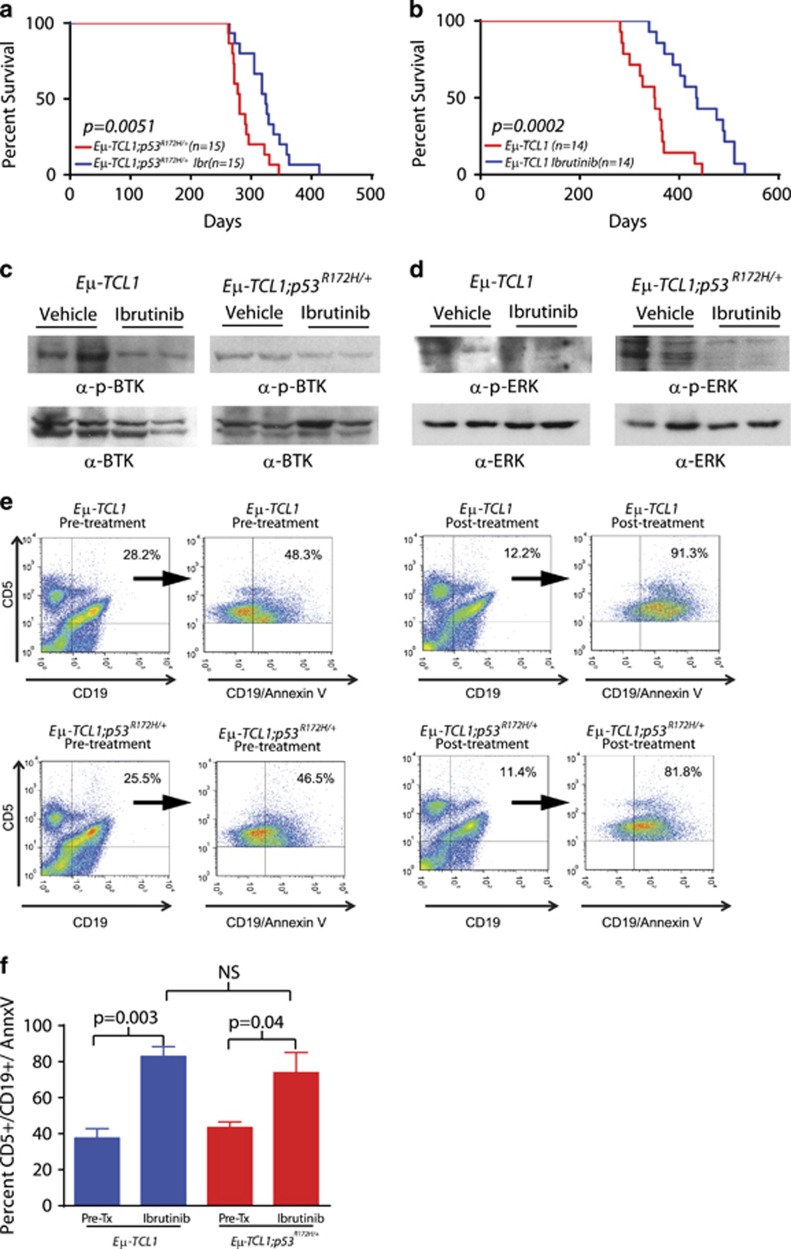
Ibrutinib prolongs survival and activates downstream pathways independent of p53. (**a**) Kaplan–Meier curves of vehicle- and ibrutinib-treated *Eμ-TCL1*;*p53*^*R172H*/+^ mice. (**b**) Kaplan–Meier curves of vehicle- and ibrutinib-treated *Eμ-TCL1* mice. (**c**) Western blot of total BTK and phospho-BTK-(Tyr223) levels in spleens from short-term ibrutinib-treated *Eμ-TCL1 and Eμ-TCL1;p53*^*R172H*/+^ mice. (**d**) Western blot of total ERK and phospho-ERK (Thr202/Tyr204) levels in spleens from short-term ibrutinib-treated *Eμ-TCL1* and *Eμ-TCL1;p53*^*R172H*/+^ mice. (**e**) Flow cytometry data demonstrating CD5^+^/CD19^+^/AnnexinV^+^ cells in the peripheral blood of *Eμ-TCL1;p53*^*R172H/+*^ and *Eμ-TCL1* mice pre and post short-term ibrutinib treatment. Cells were initially gated by their expression of CD5, CD19 and CD5/CD19 and then further analyzed by their uptake of Annexin V. (**f**) Bar graph representing the percent of CD5^+^/CD19^+^/AnnexinV^+^ cells in the peripheral blood of *Eμ-TCL1;p53*^*R172H/+*^ (*n=*4) and *Eμ-TCL1* (*n=*4) mice pre- and post-ibrutinib treatment.

To determine the efficacy of ibrutinib in p53-mutant CLL, cohorts of *Eμ-TCL1;p53*^*R172H*^ and *Eμ-TCL1* mice were treated daily with 25 mg/kg of ibrutinib via oral gavage beginning at 8 months of age. This time point is clinically relevant, as it has been previously shown to represent a time point at which *Eμ-TCL1* mice are expected to have bone marrow involvement leading to cytopenias and organomegaly.^10^ In addition, these events recapitulate a diagnostic point at which many patients with CLL begin therapeutic treatment. Ibrutinib treatment resulted in a significant survival benefit in both cohorts when compared with vehicle-treated mice (435 versus 350 days for *Eμ-TCL1* and 324 versus 281 days for *Eμ-TCL1;p53*^*R172H*/+^, Figures 3a and b, *P=*0.0002 and *P=*0.0051; respectively). Thus, the observed 19% (80 day) improvement in survival in the *Eμ-TCL1* mice and the 13% (40 day) increase in survival in the *Eμ-TCL1;p53*^*R172H*/+^ mice following treatment suggests that ibrutinib is effective in improving outcomes, even when p53 is mutated.

We next evaluated the acute inhibitory effect that ibrutinib has on the BTK pathway and cellular responses when p53 is mutated. Here, treatment-naive *Eμ-TCL1* and *Eμ-TCL1;p53*^*R172H*/+^ mice were exposed to ibrutinib or vehicle for 5 consecutive days. Western blot analyses revealed a decrease in both BTK- and ERK-phosphorylation in tumor burdened spleens from both *Eμ-TCL1* and *Eμ-TCL1;p53*^*R172H*/+^ mice (Figures 3c and d), illustrating the robust downstream inhibitory effect that ibrutinib has on the BCR signaling pathway regardless of p53 status. Next, we assessed the impact that ibrutinib has on malignant cells from *Eμ-TCL1* and *Eμ-TCL1;p53*^*R172H*/+^ mice. Using flow cytometry, we observed a significant increase in ibrutinib-mediated apoptosis in both *Eμ-TCL1* and *Eμ-TCL1;p53*^*R172H*/+^ mice, as determined by CD5^+^/CD19^+^/AnnexinV^+^ B-cells (Figures 3e and f). Furthermore, this increased apoptosis directly correlated with a reduction in CD5^+^/CD19^+^ B-cells in both *Eμ-TCL1* and *Eμ-TCL1;p53*^*R172H*/+^ mice (Supplementary Figure 1). These results suggest that ibrutinib-mediated inhibition of the BTK pathway is sufficient to reduce B-cell viability regardless of p53 status.

Given the observation that a fully intact p53 pathway is not required for ibrutinib's efficacy, and that BTK- and/or ERK-downstream targets are likely critical mediators of ibrutinib activity, we next isolated malignant splenic B-cells from treatment-naive *Eμ-TCL1* and *Eμ-TCL1;p53*^*R172H*/+^ mice and treated these cells with ibrutinib or vehicle for 24 h and performed RNA-Seq analyses. In contrast to the highly significant changes in gene expression and pathway alterations observed in the B-cells from the untreated *Eμ-TCL1* and *Eμ-TCL1;p53*^*R172H*/+^ mice, gene set enrichment analysis revealed scant expression differences between treated *Eμ-TCL1* and *Eμ-TCL1;p53*^*R172H*/+^ malignant B-cells, with no pathway differences reaching statistical significance (Supplementary Figure 2A). There were, however, expression differences in individual genes implicated in B-cell signaling. Thus, to examine potential expression changes in *Eμ-TCL1;p53*^*R172H*/+^ mice that may contribute to the ibrutinib-dependent increase in survival, we examined the expression of individual genes involved in BTK signaling pathways identified in these samples. To this end, we isolated malignant splenic B-cells from *Eμ-TCL1;p53*^*R172H*/+^ mice that had been treated with ibrutinib for 5 days. Here, we observed a significant upregulation in the receptor-mediated anti-proliferative and pro-apoptotic genes, *MAP3K15* and *PLA2G*, in tumor burdened lymph nodes and spleens of *Eμ-TCL1;p53*^*R172H*/+^ mice compared with vehicle treatment (Supplementary Figure 2B). Together, these results indicate that ibrutinib functions independently of the p53 pathway and suggest ibrutinib may be an effective treatment modality when p53 is mutated in CLL.

### Ibrutinib treatment does not place selective pressure on the *Trp53* locus

**Figure 4:**
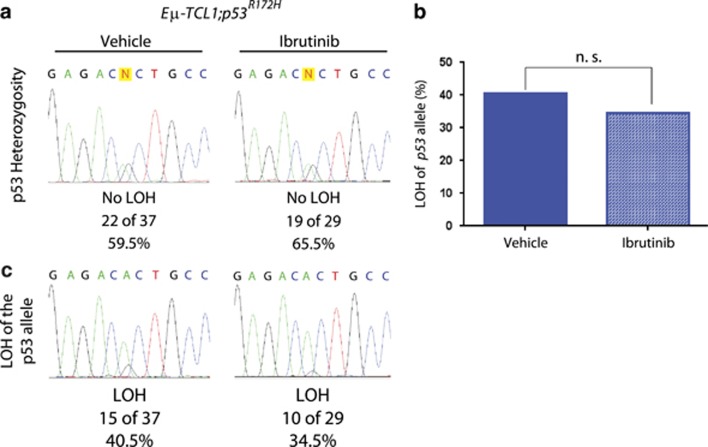
Ibrutinib does not induce LOH of the remaining wild-type *Trp53* allele compared with vehicle treatment. (**a**) LOH at the *Trp53* locus in B-cells from tumor burdened spleens and lymph nodes from vehicle- and ibrutinib-treated *Eμ-TCL1;p53*^*R172H*/+^ mice. (**b**) Percentage of LOH at the *Trp53* locus in spleens and lymph nodes from tumor burdened *Eμ-TCL1;p53*^*R172H*/+^ mice treated with ibrutinib (*n=*29) or vehicle (*n=*37).

Previous reports have demonstrated that genotoxic chemotherapy increases the selective pressure on malignant clones to lose key tumor suppressor genes through LOH.^5, 12, 19^ In CLL, patients with underlying loss or mutation of one *TP53* allele may selectively undergo a clonal selection for loss of the remaining wild-type allele following the use of chemoimmunotherapy, leading to reduced survival. To examine ibrutinib's impact on the *Trp53* locus, we evaluated LOH of the remaining wild-type *Trp53* allele in diseased spleens and lymph nodes isolated from moribund *Eμ-TCL1;p53*^*R172H*/+^ mice following vehicle or long-term ibrutinib treatment (Figure 4a). Comparison of treated and untreated spleens and lymph nodes from tumor burdened *TCL1;p53*^*R172H*/+^ mice revealed that ibrutinib did not significantly alter LOH of the wild-type *Trp53* allele (Figure 4b). This result suggests that ibrutinib does not place undue selective pressure on deleting the remaining wild-type *Trp53* allele and suggests that patients with a mutant *TP53* allele may benefit from ibrutinib-dependent inhibition of the BCR oncogenic signaling without experiencing LOH.

## Discussion

Chemoimmunotherapies have allowed many patients with CLL to achieve durable responses.^20^ However, there remains a significant subset of CLL patients—both in the *de novo* and refractory setting—who do not respond or rapidly relapse following initial standard chemoimmunotherapy-based regimens. These poor responses are often due to underlying genetic alterations that directly impact cellular functions in malignant cells. The most deleterious alterations are ones that directly ablate the p53 pathway (for example, *17p* deletions or somatic mutations in the *TP53* gene).^5^ Although deletion of the *17p* locus is routinely analyzed at diagnosis using FISH, identification of CLL patients harboring more subtle but equally debilitating p53 pathway alterations (for example, inactivating p53 mutations) remains challenging due to a lack of rapid and cost-effective diagnostic tools. This inability to reasonably identify p53-mutant CLL at diagnosis limits our understanding of how p53 mutations impact therapeutic responses and outcomes, and challenges our ability to design adequate clinical trials for this patient population. Given that recent clinical studies have suggested that p53 mutations may be present in ~10% of newly diagnosed cases,^3, 4, 5, 12^ there is a great need to identify targeted therapies that do not include genotoxic-based modalities for CLL patients. To address these issues, we developed a preclinical *in vivo* CLL model system that recapitulates the genetics of this high-risk and greatly understudied p53-mutant patient population.

Over the last decade, a significant amount of CLL research has focused on genetic alterations that predict for poor therapeutic responses and outcomes. Although these studies have provided evidence for the importance of *TP53* gene loss in the pathogenesis of CLL, they have often lacked the ability to determine the impact of specific p53 mutations on CLL progression. In this study, we observed that one of the most common p53 mutations in patients with CLL hastened the onset of leukemogenesis and shortened overall survival. As evidenced by our RNA-Seq analyses, a substantial portion of these differences can likely be attributed to the numerous changes in p53-dependent pathways between untreated *Eμ-TCL1* and *Eμ-TCL1;p53*^*R172H*/+^ mice. However, with the inclusion of ibrutinib, these *Eμ-TCL1;p53*^*R172H*/+^ mice obtained a significant increase in survival and these p53-dependent differences in expression were significantly diminished. This suggests that ibrutinib is sufficient to suppress p53-mediated expression differences between *Eμ-TCL1* and *Eμ-TCL1;p53*^*R172H*/+^ leukemias. This point is critical for patients with CLL harboring p53 mutations, as it suggests that ibrutinib (and potentially other BTK-inhibitors) will be extremely effective in treating CLL when the p53 pathway is not fully functional.

The observation that ibrutinib delays leukemic burden and improves overall survival in p53-mutant CLL, independent of a fully functional p53 pathway, is also important in the context of patients that potentially carry a single *17p* deletion (haploinsufficiency) or a heterozygous mutation (for example, p53^R175H/+^ in humans or p53^R172H/+^ in mice) as traditional genotoxic chemotherapeutic regimens are thought to place selective mutational pressure on the remaining wild-type allele to be deleted.^12^ This notion is further supported by studies in acute leukemias that have recently suggested that cytotoxic agents may allow for the selection of more aggressive subclones that are resistant to genotoxic chemotherapy.^21^ However, in contrast to genotoxic agents, the use of ibrutinib did not appear to cause such changes, as we did not observe a significant LOH of the wild-type *Trp53* gene in leukemias from *Eμ-TCL1;p53*^*R172H*/+^ mice. In fact, the percentage of LOH in *Eμ-TCL1;p53*^*R172H*/+^ leukemias was strikingly similar regardless of treatment status, suggesting ibrutinib does not promote a mutant p53-mediated escape mechanism. Given that the p53 mutational status of *de novo* CLL patients is often unknown, this finding indicates that ibrutinib may represent an excellent frontline treatment strategy for many CLL patients as: (1) it does not require a fully functional p53 pathway to be efficacious, and (2) this treatment does not appear to select for p53-mutant subpopulations when compared with vehicle treatment. Furthermore, given the fact that ibrutinib-based therapy did not directly increase LOH of the wild-type *Trp53* allele in treated *Eμ-TCL1;p53*^*R172H*/+^ leukemias compared with untreated *Eμ-TCL1;p53*^*R172H*/+^ leukemias, these data also imply that the p53 pathway will remain partially active in these patients. This is a salient point, as it suggests combinational approaches that may include ibrutinib plus novel apoptotic activators (for example, ABT-199) or immunomodulators (for example, lenalidomide) may offer a significant benefit for patients who harbor a single p53 alteration. These types of simultaneous anti-proliferation and pro-apoptotic treatment modalities may provide a deeper remission^22^ and potentially lead to eradication of the high-risk CLL.

Even though ibrutinib has been shown to be efficacious in difficult to treat B-cell malignancies, such as salvage therapy in mantle cell lymphoma and 17p- CLL, many of these patients will eventually relapse with ibrutinib-refractory disease.^23, 24^ Often these relapses are due to critical mutations in the BTK pathway that inhibit ibrutinib binding to the BTK protein (C481S)^24^ or activating mutations in (PLCγ2)^24^ that bypass the need for BTK's activity. Thus, in the very near-term, alternative treatment strategies will be needed for these patients. Given our observation that ibrutinib does not place significant pressure on the p53 pathway, these refractory patients, who still maintain at least one copy of p53, may still receive a substantial benefit from genotoxic and/or cytotoxic agents, like fludarabine and cyclophosphamide in a primary or secondary salvage setting.

In summary, our results indicate that ibrutinib is effective in delaying leukemic progression and increasing survival in *Eμ-TCL1* mice that carry a p53 mutation. This result is critical for CLL patients with mutant p53 (or even a *17p* deletion), as they indicate that ibrutinib does not require a fully functional p53 response for its efficacy, and thus primarily exerts its effects through mechanisms outside the p53 pathway. Similarly, our examination of the dynamic loss of the remaining wild-type *Trp53* allele revealed that ibrutinib did not force LOH of the remaining wild-type *Trp53* allele, indicating that this pathway may be amenable for activation with either combinational therapies or in an ibrutinib-resistant setting. Critically, these data demonstrate the potent effect of BTK-inhibition in B-CLL and, more importantly, provide evidence that ibrutinib is an effective treatment for aggressive forms of B-CLL with *TP53* mutation and potentially chemo-resistant refractory disease.
