# Supplementary figures and images for: p53-independent ibrutinib responses in an Eμ-TCL1 mouse model demonstrates efficacy in high-risk CLL
Source: Blood Cancer J. 2016 Jun 10;6(6):e434–. doi: 10.1038/bcj.2016.41 (PMC5141356; doi:10.1038/bcj.2016.41)

Supplemental Figure 1

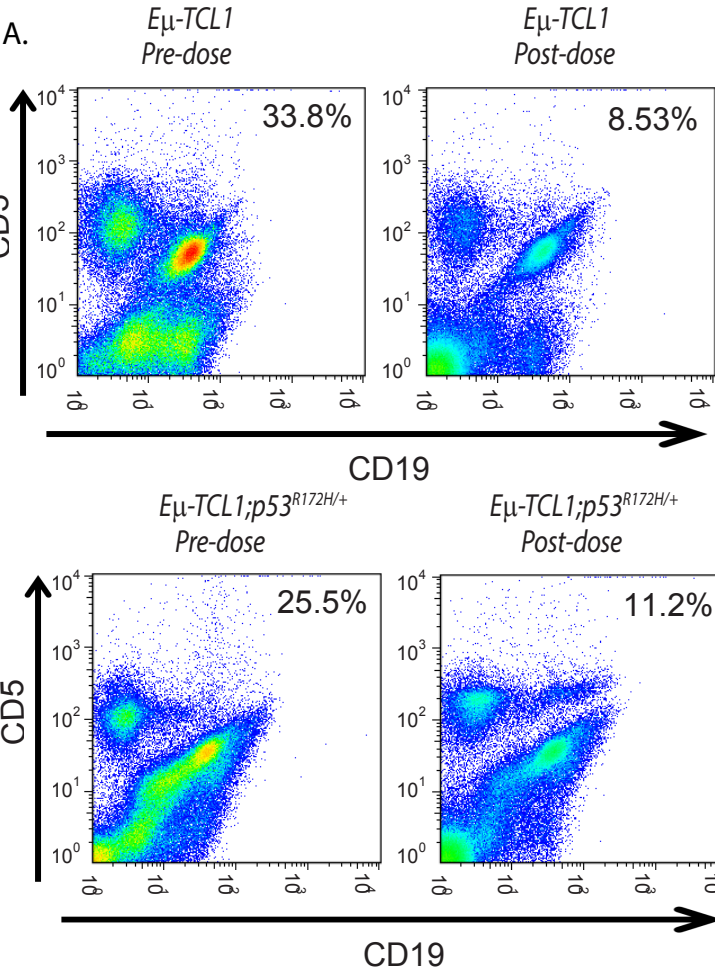

Supplement: Supplementary Figure 1 [file bcj201641x1.pdf]

Supplemental Figure 2

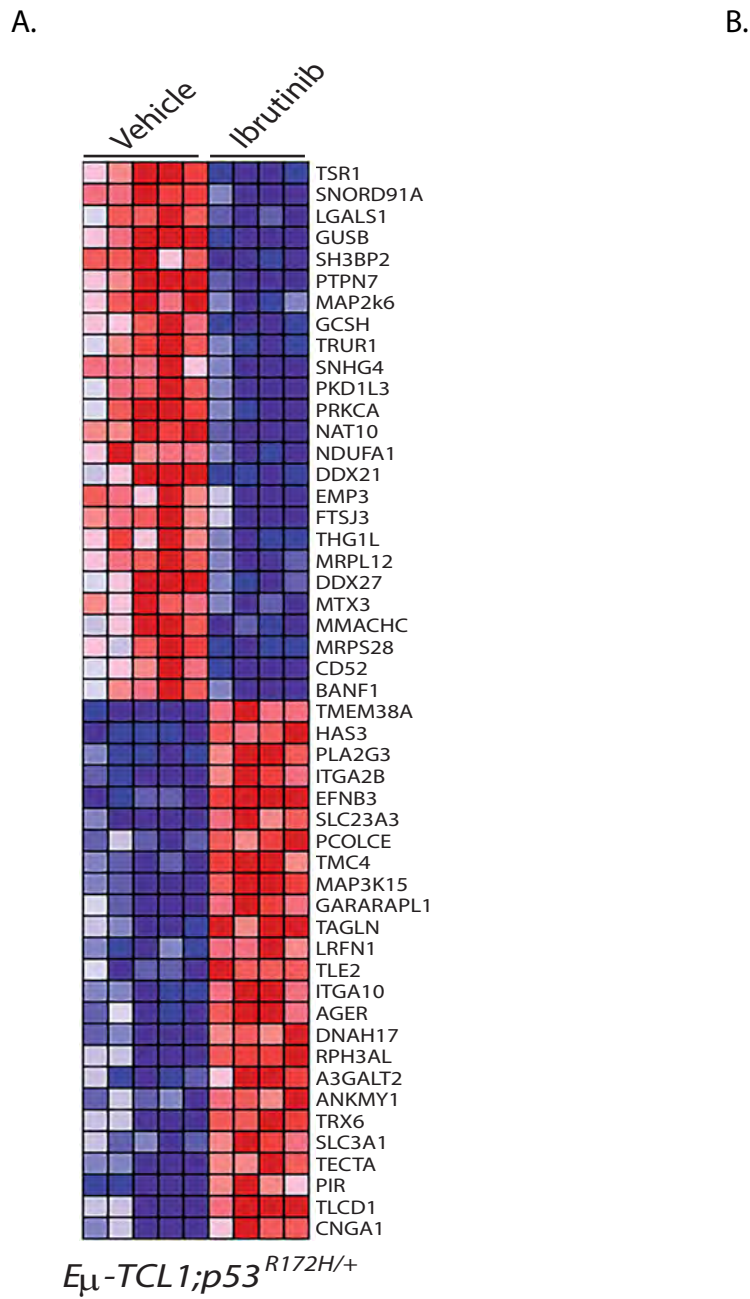

Supplement: Supplementary Figure 2 [file bcj201641x2.pdf]
