# Supplementary material for: p53-independent ibrutinib responses in an Eμ-TCL1 mouse model demonstrates efficacy in high-risk CLL
Source: Blood Cancer J. 2016 Jun 10;6(6):e434–. doi: 10.1038/bcj.2016.41 (PMC5141356; doi:10.1038/bcj.2016.41)
Supplement: Supplementary Figure Legends [file bcj201641x3.doc]

**Supplemental Figure Legends**

**Supplemental Figure 1.**

(A) Flow cytometric analysis of CD5+/CD19+ cells following short-term ibrutinib treatment in the peripheral blood of *Eµ-TCL1 and Eµ-TCL1;p53^R172H^*^/+^ mice.

**Supplemental Figure 2.**

(A) Heat map of hierarchical gene expression profile of differentially expressed genes after ibrutinib-treatment in B-cells from *Eµ-TCL1 and Eµ-TCL1;p53^R172H^*^/+^ mice.

(B) qRT-PCR of *MAP3K15* and *PLA2G* expression in B-cells from ibrutinib-treated *Eµ-TCL1;p53^R172H/+^*spleens.
