# Supplementary material for: p53-independent ibrutinib responses in an Eμ-TCL1 mouse model demonstrates efficacy in high-risk CLL
Source: Blood Cancer J. 2016 Jun 10;6(6):e434–. doi: 10.1038/bcj.2016.41 (PMC5141356; doi:10.1038/bcj.2016.41)
Supplement: Supplementary Information [file bcj201641x4.doc]

**Supplemental Methods**

**Short term ibrutinib treatment**

Eμ-TCL1 and Eμ-TCL1;p53^R172H/+^ cohorts were monitored daily for signs of disease progression. At eight months of age, mice were weighed and treated with ibrutinib dissolved in double distilled water (25mg/kg/day, Selleck Chemicals) via oral gavage for 5 consecutive days. Mice were sacrificed 24 hours after final treatment and diseased organs were harvested. Tissues were examined by flow cytometric analysis or flash frozen for molecular analyses.

**Flow Cytometry**

Flow cytometry was performed using cells isolated from the peripheral blood of Eμ-TCL1 and Eμ-TCL1;p53^R172H/+^ mice. Peripheral blood was collected from both untreated and short-term treated animals. Briefly, erythrocytes from the peripheral blood were lysed using red blood cell-lysis buffer (Becton Dickenson) and approximately 1x10^6^ mononuclear cells were washed to create single cell suspensions. Cells were re-suspended in binding buffer (Becton Dickenson) and incubated (30 minutes at 4°C) with α−CD19-PECy5 and α−CD5-PECy7 antibodies. Approximately 100,000 cells were acquired per sample using the Gallios flow-cytometer and software (Beckman Coulter). All data were analyzed with FlowJo software (version 8.8.6; TreeStar, Ashland, OR, USA).

**RNA-Sequencing**

Malignant B-cells were isolated from leukemic burdened spleens of Eμ-TCL1;p53^R172H/+^ mice by mechanical disruption. Isolated lymphocytes were placed in culture media (RPMI 1640 supplemented with FBS and Pen/Strep) and immediately treated with either ibrutinib (5µM) or vehicle (DMSO). Twenty-four hours post-treatment, cells were collected and total RNA was isolated. Genotypic- and treatment-specific changes in RNA expression were determined by RNA sequencing on an Illumina platform in the MD Anderson Genotyping Core Facility. Gene sets were analyzed by the MD Anderson Bioinformatics Core. Raw sequence data is available from the NIH/GEO database: <http://www.ncbi.nlm.nih.gov/geo/query/acc.cgi?acc=GSE76183>.

**Real-time RT-PCR**

Malignant lymphoid cells were isolated from the spleens of tumor burdened Eμ-TCL1 and Eμ-TCL1;p53^R172H/+^ mice. Total RNA was extracted and purified using Trizol followed by DNAase-I treatment. First-strand synthesis from 1µg of total RNA was performed using iScript-RT (BioRad). The resulting complementary DNAs (cDNA) were amplified by using primers sets *MAP3K15*: Forward; 5’-TGATGAACTGGCGAAAGAGTTG-3’ and reverse: 5’- ATGATGATGTCCGATGTCAGAAC-3’ and *PLGA2*: Forward; 5’- AGAGACCACAGGGCCATTAAG-3’ and reverse: 5’-GCTGTAGAATGACATGGTGCT-3’ and *Rplp0*^1^*.* All assays were performed in triplicate and changes in expression were compared using the Pfaffl method^2^ by comparing expression changes between target genes and the housekeeping control *Rplp0.*

**Supplemental References**

1. Zhang X, Pageon L, Post SM. Impact of the Mdm2(SNP309-G) allele on a murine model of colorectal cancer. *Oncogene*. 2015;34(33):4412-4420.

2. Pfaffl MW. A new mathematical model for relative quantification in real-time RT-PCR. *Nucleic Acids Res*. 2001;29(9):e45.
